# The Hospital. Nursing Section

**Published:** 1901-10-26

**Authors:** 


### The Hospital.

### Nursing Section.

Contributions for this Section of "THE HOSPITAL" should be addressed to the EDITOR, "THE HOSPITAL" NURSING SECTION, 28 & 29 Southampton Street, Strand, London, W.C.

No. 787.—VOL. XXXI. *SATURDAY, OCTOBER* 26, 1901.

### Notes on News from the Nursing World.

### THE NURSING ESTABLISHMENT OF THE SOUTH AFRICAN CONSTABULARY.

WITH reference to the Nursing Establishment of the South African Constabulary, General Baden-Powell has kindly favoured us with the following information :—"We take the nurses on a three years' engagement at £120 a year, with rations, quarters, laundry, etc., found, as well as hat, cape, belt, and great coat. The dress consists of brown holland dress, white apron, dark green cape, cape of Army Nursing pattern, and cowboy hat and khaki ulster great coat. We have at present six hospitals of 100 beds, and several detention hospitals of 10 beds in different parts of the country. Candidates for appointments should apply to Colonel Beevor, P.M.O., S.A.C., Johannesburg." Many of our readers will, we are sure, be glad to be in possession of these authoritative facts.

### A STAB IN THE DARK.

VAGUE assertions are, as a rule, hardly worth notice. But the *Freeman's Journal* in its anxiety to deter Irish girls from seeking to enter English hospitals as probationers, commits itself to the statement that "one of the greatest and richest of London institutions has even refused its nurses meat more than three or four times a week." For the sake of the nurses who are alleged to be thus underfed, as well as to free from suspicion great and rich London institutions which it is not intended to reflect upon, we ask our contemporary to name the hospital it alludes to. We shall then be in a position to ascertain the basis of an accusation which, as it at present stands, is a stab in the dark that may injure the innocent as well as the guilty—if guilty there be, and in any case will injure the Irish girls who believe the story.

### AN UNFEELING NURSE.

THE superintendent nurse at Skipton Workhouse Infirmary has mistaken her vocation. At the usual meeting of the Skipton Board of Guardians Colonel Maude stated that the wife of an old man had recently been admitted to the infirmary in a precarious condition of health. The husband, who was approaching 80 years of age, subsequently sent a stamped envelope to the infirmary asking to be informed of the condition of his wife, but received no reply. He afterwards journeyed to Skipton, and called at the infirmary, where he saw the superintendent nurse, and was told that his wife had been dead several days. "When asked why he had not been informed, the nurse, who admitted having received the stamped envelope, replied that it was not her duty to write letters. The old man was greatly affected at the tidings, and had since declared that he would rather die on the road side than enter the union." The board strongly condemned the want of sympathy shown by the superintendent nurse, and might have taken action in the matter, but the poor old man himself having asked that she should only be warned, his request was acceded to. His generosity contrasts favourably with the unfeeling conduct of the superintendent nurse, who may, nevertheless, find her position too uncomfortable to remain in it.

### A QUESTION OF PRINCIPLE AT BRISTOL INFIRMARY.

THE accuracy of the following facts is vouched for by a correspondent whose knowledge should be unimpeachable :—"An untrained nurse was transferred from the workhouse to the Stapleton Union Infirmary, Bristol, upwards of two years ago. She was appointed assistant nurse to the aged sick and had charge of the wards for some time. Lately she has been transferred to the male block and has charge of a ward in which there are medical and surgical cases. She has done no actual probationer's work nor has she been treated in any way as one. Neither has she done night duty. She has had assistant nurses' salary, rising from £24 to £28, while the probationers rise from £10 to £20. Personally, the nurses have no ill feeling towards her, but they all think it unfair (as she did not begin at the beginning and go through her training) for her to have a three years' certificate. The medical officer says that she will get one." We trust that the medical officer will prove to be mistaken. It will become hopeless to attempt to improve nursing in workhouse infirmaries if certificates are to be accepted by the Local Government Board in cases where the recipients have not gone through the ordinary training.

### ENTERTAINMENT TO LIVERPOOL NURSES.

LAST week the Lord Mayor and Lady Mayoress of Liverpool entertained a number of nurses in the Town Hall on two successive evenings. Invitations were sent to the nurses at the Royal Infirmary, David Lewis Northern Hospital, Royal Southern, Stanley, and Hahnemann Hospitals, Brownlow Hill, Toxteth Park, and West Derby Workhouse Hospitals, the Infirmary for Children, Hospital for Women, Ladies' Lying-in Hospital, Queen Victoria District Nursing Institution, the Nurses' Homes at Oxford Street, Erskine Street, and Catherine Street, besides many of the other public institutions. The invitations would have been extended to the nurses of the city hospitals; but, as the Lord Mayor explained, he was afraid that the public might be alarmed and dread possible infection. The nurses from the Consumption Hospital donned their grey and white uniform, and the graceful mantilla-like headdresses of Shaw Street Hospital were conspicuous. All the nurses from the Southern Hospital wore pretty badges composed of red roses, lilies of the valley, and maidenhair fern. Dancing took place in the large ball-room, and though many men were present, they were not numerous enough to go round, and many of the nurses, therefore, more in sorrow than in anger, danced with each other. A delightful chamber concert was a feature of the entertainment, which was immensely appreciated by all the guests.

### POOR LAW NURSING IN IRELAND.

There is another hopeful sign for the future of poor law nursing in Ireland. Surgeon M'Ardle, at the annual dinner of the medical staff and past students of St. Vincent's Hospital, Dublin, last week, having declared his intention to support the Irish Local Government Board "through thick and thin" in its demand that all nurses who have to face great responsibilities should be trained in great hospitals, went on to suggest the separation of poor law infirmaries from the workhouse. He contends that a clause in the Act of Parliament empowers the Local Government Board to dissociate the two institutions, and to describe the workhouse infirmary as the district hospital. He also urges that the district hospitals thus secured should be open "to all who need them." This is a large question about which there is a good deal to say. But the separation which Mr. M'Ardle recommends could not fail to contribute to the elevation of the nursing standard. Meanwhile, the Irish Local Government Board have advised the Armagh Board of Guardian that no sufficient facilities exist in the Union Infirmary for the training of probationers, and that the nursing staff is insufficient. The guardians have referred the matter to the "special nursing committee," who, if they are wise, will advise the abandonment of further resistance.

### DISORDER IN THE WARDS.

If it is possible for English matrons and nurses to learn a good deal from foreign hospitals, the English nurse who in our last issue gave an interesting account of a visit to the Hamburg Krankenhaus shows very conclusively that the Germans have something to learn from us. The picture of the sisters walking about the wards without noticing the bits of coal and crumbs on the floors, the empty mugs, the dressings by the bedside, is very unpleasant; while the absence of flowers and air is not less deplorable than the absence of discipline. "The Diary of a Naughty Probationer," which appears in another column this week, seems to suggest that the discipline in English hospitals is sometimes unduly severe. But it is better for both nurses and patients that it should err in that direction than that it should tend to the laxity depicted by our Hamburg contributor. Under the auspices of the ideal matron the happy mean is easily achieved.

### CHARGE OF CRUELTY AGAINST A CAMBERWELL NURSE.

A nurse who is convicted of cruelty to a patient deserves no mercy; but unless, or until, such a charge is fully substantiated, she assuredly ought to have the benefit of the doubt. But the doubt ought not to be left hanging over her head, and although the Camberwell Board of Guardians, who were invited to prosecute a nurse who is accused of cruelties to patients, did well to accept an alternative proposal to refer the question back to the visiting committee for further inquiry, this committee is bound either to prosecute or to exonerate completely. At present the accusation appears to rest mainly upon a report in the journal of the workhouse master, who states that the nurse was suspended upon certain allegations, although the doctor, the matron, and the night superintendent are said to have given her a very good character. It is true that one of the guardians makes more specific charges, and avers that the nurse forced a cup of beef-tea into the mouth of an old woman; that she threw an old inmate on the bed and said to her, "Lie there, you old pauper;" and that in another instance, she "jostled" an inmate in the corridor, though the latter was so ill that in half an hour she was dead. But as the guardian himself admitted that evidence as to these most serious charges had not been placed before the visiting committee, the necessity of a thorough investigation is clear in the interests of the patients, no less than in justice to the nurse, whose professional reputation is at stake. We are inclined to think that in similar cases a police court might often prove a better tribunal than a committee.

### NEW NURSES' HOME AT ALTRINCHAM.

There has just been opened by the Duchess of Buckingham a commodious new home for the accommodation of the nursing staff of the Altrincham Provident Dispensary and Hospital. The home had been in possession of the nurses for some weeks before the formal ceremony, and the Duchess of Buckingham had, therefore, the advantage of seeing it occupied. Her testimony is that the rooms are very comfortable, and that everything had been done in very good taste. The whole expense of acquiring and fitting up the building is £3,400, and it is naturally hoped by the managers of the hospital that the expense incurred in providing for the needs of the nurses will be met by increased contributions. Dr. Golland rightly said, at the opening function, that the art of nursing has become so much more important in recent years that the accommodation which might be sufficient for nurses of the "Sairey Gamp" type, is inadequate for the highly-trained nurses of the present day, and that it is desirable for them to have a home quite independent of the hospital, so that their leisure may be spent amid pleasant surroundings.

### PENSIONS FOR SUTHERLAND NURSES.

The sixth annual report of the Sutherland Benefit Nursing Association, of which the Duchess of Sutherland is president, shows that there are at present 13 nurses on the staff. The number of cases nursed during the year ending July 31st last was 296, an increase of 43 over the preceding year. We are glad to notice that the financial condition of the association is so satisfactory that the committee have been able to enter into an Endowment Insurance for the benefit of such nurses as may, in their opinion, merit a small pension in their old age. We hope that this is in co-operation with the Royal National Pension Fund which offers such great advantages to nursing institutions in connection with its affiliation scheme. So far, the question of pensions for district nurses has been little considered, chiefly, no doubt, because of the difficulty so often experienced in collecting enough money to defray the ordinary expenses. But we trust that the time is not very distant when it will be the rule, and not the exception, to assist a district nurse in making provision for the period when she is past work.

### THE RELIGIOUS TEST.

At a meeting of the Ecclesall Board of Guardians last week a charge nurse and a probationer for the workhouse infirmary were appointed. One of the applicants for the former position was asked by a member of the board to "what religious faith she belonged"; and a would-be probationer, having first been called upon to state whether her friends in Sheffield were "ladies or gentlemen," was subjected to a similar interogation. We agree with the guardian who protested that there should be "no religious test." The "religious faith" of a nurse no more concerns the guardians than the sex of the friends she happens to possess.

### THE METROPOLITAN ASYLUMS BOARD.

At a meeting of the Metropolitan Asylums Board on Saturday the children's committee reported that they had received a letter from the School Board for London asking whether Miss Lockington, who has been appointed as a nurse to report on the presence of ringworm in the schools of the Board, for the purpose of training, and also of observing the latest methods of the treatment of the disease. The committee stated that they had referred the letter to the Bridge School sub-committee for them to make such arrangements as they may consider necessary to comply with the School Board's request, and they had so informed that Board. The action of the committee was endorsed. The Board decided to award a gratuity of £20 to Miss Barton, who had discharged the duties of matron of the hospital ships for about eight months during the absence of Miss Wacher.

### NURSES' ACCOMMODATION AT NEWTON ABBOT.

The latest phase of the nursing question at Newton Abbot arises in regard to the sleeping accommodation and the position of the recreation room. The fact that the new master has drawn attention to the matter shows that he, at all events, appreciates the difficulty. It appears that only two of the six nurses have separate bedrooms; that the night-nurses' quarters are situated over the maternity wards, and their sleep is disturbed by the crying of the infants; and that their recreation room is in such proximity to the sick quarters that they can scarcely raise their voices —and certainly not amuse themselves, except silently —without disturbing the patients. The master has suggested the desirability of erecting a nurses' home, and the guardians have resolved to obtain information as to what is done elsewhere. We hope that this will be convincing, and that the guardians will make haste to provide a building where the nurses can not only rest but enjoy themselves when off duty without disturbing the patients.

### TRAINING IN BORDEAUX.

Nursing in France is at so low an ebb that the first efforts at improvement are especially interesting. The Protestant House of Health at Bordeaux, which has been established since 1884, now receives probationers and trains them for two years. During that time, these pupils are placed under the direction of certificated nurses, and they receive practical instruction, the theoretical knowledge being imparted by means of lectures, which are given during the first year for eight hours, and during the second for twelve hours, in every month, as well as 16 conferences in the last year. There are three preparatory examinations and another at the end of the course. The lectures are given by the lady superintendent and the doctors belonging to the hospital, and, occasionally, by strangers. There are 68 beds, and the hospital receives eight probationers, besides several paying pupils. In addition, there are a number of out-probationers who can, if they wish, follow the same course as those who live in the house, and go through the same examinations, ultimately receiving the same certificate. The probationers are on duty from 6·30 A.M. till 9 P.M. with two hours off duty daily, one day a month and one month a year. Those who take night duty from 8 P.M. to 8 A.M. have the following day free for rest and exercise. All probationers must belong to the Protestant religion, be between the ages of 21 and 35, having received a good education and possessing good health and a guarantee of moral character. Since May, 1901, the House of Health has been under the charge of Dr. Anna Hamilton.

### A LANCASHIRE NURSING ASSOCIATION.

In the annual report of the Garstang and District Nursing Association the committee request that "the nurses may not be called out on Sunday except in urgent cases." This is reasonable, especially as in the whole of the very large district only two nurses are engaged. But as the balance of income—thanks largely to the proceeds of a sale of work—exceeded by £136 the expenditure, even after the sum of £200 had been invested in railway stock, it might be practicable to appoint a third. The lectures given by Nurse Dell on Sick Nursing appear to have been greatly appreciated.

### RESIGNATION OF THE MATRON OF STIRLING INFIRMARY.

The important post of matron of the Stirling Royal Infirmary has become vacant by the resignation of Miss Falconer, who has held it since the opening of the institution, more than twenty-seven years ago. The chairman of directors, in announcing Miss Falconer's retirement, said that during the long period she had been with them she had discharged her onerous duties in a most devoted manner, and all his colleagues bore witness to her ability and faithful services. She collected upwards of £370 in a month for the furnishing of the new wing of the hospital, and at her suggestion endowed beds were initiated. The vacant appointment is worth £60 per annum, and it is expected that a selection will be made on November 4th.

### THE CURETON TESTIMONIAL.

It has been decided not to have any formal presentation to Miss Cureton, the late matron of Addenbrooke's Hospital, Cambridge. The chairman of the committee will present to Miss Cureton an address on behalf of all the subscribers, with a cheque. As intimated in another column, the testimonial account will close on Saturday, and a final meeting of the committee is called for Monday.

### SHORT ITEMS.

The *Britannic* arrived at Southampton from South Africa on Friday last, with Nursing Sisters Boyd-King, L. J. Attres (A.N.S.R.), M. Steel and — Fraser (N.S.W.A.N.S.) on board. All return to South Africa at the expiration of leave.—Nursing Sister Lillian Smith, who is serving in South Africa, was discharged from hospital to duty in the week ending September 29th.—The *Mohawk* arrived at Southampton from South Africa on Monday, the following being on board: Sisters Brown, M. McIntosh (both wishing to return in the same vessel), and A. W. Micall.

### Lectures to Nurses on Medicine and Medical Nursing.

By Norman Dalton, M.D. London, F.R.C.P., Physician to King's College Hospital.

### LECTURE XXIII. — DISEASES OF THE CEREBRO-SPINAL SYSTEM—DELIRIUM.

The functions of the cerebro-spinal nervous system are of three kinds, namely, mental functions, motor functions, and sensory functions. Each of these functions, when affected by disease, may become deranged in two ways, that is, they may become excited so that a kind of overaction of them occurs, or they may become lessened or lost. Consequently we have to consider six conditions of the cerebro-spinal nervous system. First, "delirium" in which the mental functions are excited, and, secondly, "coma" (that is unconsciousness), in which they become absent. It is obvious that, between these extremes, there are minor degrees of mental excitement, falling short of actual delirium, on the one hand; and conditions of mental obtuseness, short of complete unconsciousness, on the other. Thirdly, there are "convulsions" or "spasms" in which the motor functions are excited, and, fourthly, "paralysis," in which they are lost. If the power of movement is diminished without being lost, the word "paresis" is applied to the condition. Fifthly, we have to consider "pain," in which the sensory functions are excited; and, sixthly, "anæsthesia," in which they are lost. Increased sensitiveness to the touch, short of actual pain, is called "hyper-æsthesia;" and, on the other hand, we often speak of "numbness" when the power of feeling is diminished without being quite lost. If we consider the above-mentioned six morbid conditions in relation to diseases of the brain and spinal cord, we may say that, practically, delirium and coma only occur in affections of the brain or when poisoned blood circulates through the brain. Paralysis and spasms occur both in diseases of the brain and of the cord, though the spasms are of a different nature in each case. Pain also occurs both in brain disease and in spinal disease; but anæsthesia, if permanent, is nearly always due to spinal diseases. In the above remarks the nerves themselves have been left out of consideration; but it must be remembered that spasm and paralysis, pain and anæsthesia may be due to diseases of the nerves, and not to diseases of either the brain or the spinal cord.

*Delirium.*—The greatest and most permanent disturbance of the mental functions of the brain occurs in insanity, but the nursing of the insane is a subject which can only be taught by specialists in mental diseases. Great mental derangement may be caused by tumours in certain parts of the brain and by other organic cerebral diseases, but the form of delirium which usually comes under the observation of a nurse is that which is caused by a poison circulating in the blood-vessels of the brain. The commonest poisons which so affect the brain are those which are generated in the body during the course of typhoid fever, or of typhus and other specific fevers; but the poisons of uræmia, of acute yellow atrophy of the liver and of many non-febrile diseases may produce the same disturbances of the mind. In a typically severe case of "typhoid fever" delirium begins towards the close of the second week and is preceded by worrying dreams from which the patient wakes in a dazed condition. In the early stages he soon throws off the influence of his dream and realises his surroundings; but, after a day or two more, his dreams persist after he is awake; in other words he becomes delirious and talks and acts incoherently. Very often he makes an effort to get out of bed, not unfrequently under the idea that he has an important engagement to keep. Now, in typhoid fever, this is most dangerous, because movement, especially in the erect posture, is very apt to cause perforation of the bowels. Getting out of bed must, therefore, be prevented at all costs. Persuasion will often induce a patient to remain in bed or to return to it, if he has escaped, particularly if the nurse be successful in catching the patient's leading idea and can satisfy his mind that his urgent appointment can be arranged for or that his anxieties are unfounded. Prevention is better than cure and it is easier to keep a patient in bed than to get him to return to it. Consequently a delirious patient must be closely watched; and, if an attempt to get out of bed has to be met by gently exerted force, the sheets should be pinned across his shoulders. Fortunately the delirium of typhoid occurs, usually, late in the disease when the patient is already exhausted by 14 days of fever and the exhibition of *force majeure* is not often called for. But in typhus fever the delirium comes on earlier, before the patient has become extremely weak, and very strong measures may be necessary to keep him in bed. Fortunately in typhus there is no fear of perforation and the only risks the patient runs by wandering about the ward are those of an increase of his exhaustion and, possibly, the effect of exposure to cold. The weakness of the typhoid patient causes him to limit his performances to talking incoherently, to picking at surrounding objects and to making uncertain efforts to get out of bed, while typhus patients are more likely to shout and struggle. Nurses must, however, be prepared to meet with exceptions to these types.

The worst cases of delirium with which a nurse may have to deal are cases of "delirium tremens." This affection occurs as a consequence of constant alcoholic excess. It may occur after months of continual "soaking," or during a drinking bout, prolonged over several days. But it frequently breaks out, in habitual tipplers, after a simple accident, or an operation, or during pneumonia or some other specific disease. So that when in charge of what appears to be a simple case, if a nurse be told that the patient is addicted to drink she must not be surprised at the sudden setting in of delirium tremens in its most acute form. It is impossible to describe all the vagaries of a patient suffering from delirium tremens. He may be violent and noisy, or quiet and muttering. At intervals he may appear quite rational. The great characteristics of the disease are that the limbs tremble and that the patient sees "spectra," *i.e.*, he may believe that he sees beetles, rats, snakes, or monsters which haunt or threaten him. Delirium which takes this form is nearly always the result of alcohol, and, if alone with her patient, a nurse must carefully note anything he may say in his wandering which might indicate that he is seeing "spectra." In a bad case the terror of the patient is most distressing to witness. He may at one time entreat the attendant most piteously for help, and at another abuse her most foully. If not controlled he may assault the bystanders, or may commit suicide by any means which presents itself. Nurses must remember above all things that a patient with delirium tremens can never be trusted. He may appear perfectly rational, may beg to be released from his strait-jacket, and may promise to be perfectly quiet; but the moment he is loosed he may do violence to himself or others. If uncomplicated by pneumonia, Bright's disease, or other organic affection, patients with delirium tremens often get well if they can be fed and made to sleep. The latter object can be effected by hypodermic injections, and the former, at the worst, by a tube passed through the nose. But the nurse may by tact succeed in getting her patient to take food by the mouth, which is a great desideratum.

In belladonna poisoning patients frequently become delirious. They may be violent, and like the patient with delirium tremens they cannot be trusted. I have known a patient who distracted the nurse's attention and sprang from his bed on to the window sill. Fortunately there was a ledge outside and his nightdress was seized before he jumped down.

### Diary of a Naughty Probationer.

FIRST WEEK IN HOSPITAL.

Tuesday Night.—Here I am in hospital at last; it does feel queer. What miles of stairs I had to climb to reach the "bit" of a bedroom allotted to me. Met a large person in the dormitory corridor shortly after my arrival. Thought she must be the cook; made inquiries, and find she is a very important person called "home sister." Shall have to stifle my inclinations, I suppose, to call her "cook." Have spent nearly an hour trying to fix a very ugly cap on my head as a "trial trip" for to-morrow morning.

Wednesday, 9.30 p.m.—Have had an awful day. Got up about 5 a.m. so as to be in plenty of time for breakfast at 6.30! Was sent to a male medical ward. Staff nurse very short in her manner. Kept telling me all the time to "hurry up." I said "Good morning, sister," when that lady entered the ward, but received only a stony stare from her dark eyes. Was told afterwards I should not speak to such a mighty personage as a sister until she speaks to me; shan't make that mistake again. Feel too sleepy to write more.

Thursday, 9 p.m.—Don't know what I should do without this little book, can say just what I like in it, am not allowed almost to speak in the ward; staff nurse keeps saying, "Nurse, you must not talk," or "Nurse, you must not laugh." Hope my tongue won't get atrophied! Was sent for at 10 a.m. to go to the matron's office; felt I would nearly as soon take the train home. My fellow probationer slapped me on the back and told me "to go to my doom like a man;" that did not cheer me much. Got inside the office somehow; heard a voice say kindly, "Good morning, nurse." I whispered "Good morning," and ventured to look up at the great personage, and oh! what a sense of relief came over me. She was beautiful, and looked perfectly charming; she talked to me quietly for a few minutes, but I was so engrossed admiring her I hardly heard what she said. I left the office quite jubilant; here at least I felt I would find sympathy and justice; my fellow probationers seemed rather surprised at my increased spirits when I returned to the ward. Broke a specimen glass in the evening; confessed to sister. She talked to me on the enormity of such carelessness for at least 15 minutes; felt at the conclusion that never was woman so wicked as I. Good night, little book.

Friday, 9.15 p.m.—Have had the most extraordinary day. Was told at breakfast by night sister that I was to go to a female ward this morning, as a probationer from there was off duty ill. Such a queer old sister in this ward; she bustles in and out and runs up and down without doing any work herself. She wears her cap all down about her ears, and looks, I think, quite an old sight. She caught me reading a letter from home in the bathroom, and oh! dear, what a lecture I got on the "idleness, etc., etc., of a new probationer," I felt quite a culprit. One of the students asked me to fetch him something for the test-table, and when I brought it to him sister came running along from her table, "You must ask me for what you want; you must not talk to the probationers." Then she took me to her table and there read me another lecture on the sin of encouraging a student or doctor to speak to me on any pretext whatever. To keep me quite safe and out of mischief she set me to sew an old dressing-gown; I was made to sit just opposite her table, and from there she eyed me through her glasses. At all times I hate sewing, but to have to sew an ugly garment such as that dressing-gown was quite vexatious. Only one gleam of pleasure did I have during the day's work, sister sent me a message to matron's office, and I refreshed myself by the few minutes I had with the gracious lady there. "Are you busy in the ward to-day," she asked as she looked for the papers sister wanted. "No," I answered, "not at all, sister has put me on as 'special' to a dressing-gown." Matron looked at me with laughter in her dark brilliant eyes. "You don't enjoy sewing, then," she said when I explained the nature of the "special" duty. "No, I don't," I replied, "and I don't like the dressing-gown, and I don't like anything in the 'Queen's' Ward," I finished desperately. "That is very bad of you," she said gravely, but again I saw the flash of fun in her eyes that gave one such a delicious sensation of being understood, instead of condemned. Here comes the home sister to see that the lights are out.

Saturday, 9 p.m.—Must not write so much to-night; was reported to matron this morning by home sister for having turned on my light after she had been round. Fact was I sat writing in this book and quite forgot to undress, so had to light up after home sister went away, but she came back to see if she could catch some of us tripping, and, of course, she caught me and several others. Have had another day with Sister St. Mary in the "Queen's Ward." I do hope the sick probationer will soon be well and come back to her own work. The night nurse tells me this old sister is very "churchy." I feel reproached as I had come to the conclusion she was a wicked old person, but of course if she goes to church every morning she must be good! Had my first lesson from Sister St. Mary on dressing a bedsore; she first of all collected us round the bed—sister, staff nurse, night nurse, and two probationers—staff nurse held the patient, I held the basin of warm water, the other pro. held the powder, &c., the night nurse cut the dressing to fit, and sister put it on. I remarked afterwards to staff nurse on the gravity of the proceeding and she said, "You little stupid, do you suppose any other person fusses over a bedsore like Sister St. Mary."

Sunday, 8 p.m.—Have had really a nice day, did not get a single scolding all day. Sister St. Mary had a half-day off. Was sent off duty this evening at 6. No fear of home sister to-night; she also has a half-day off.

Monday, 9 p.m.—Another day in the female ward, sister seemed refreshed after her half-day off, and we were all made to "sit up" this morning; even the wardmaid confided to me in the privacy of the little kitchen, "Sister do seem to get crankier every day, that she do." My fellow pro. had a bad time; we all thought sister was safely away at lunch, but back she came unexpectedly, like a whirlwind, swept me out of the kitchen, where I was inoffensively drinking a glass of milk, whisked me into the ward "to find something to do," but there her interest in me ended for the time. At the far end of the ward, in direct line of the eagle eye at the door, stood Nurse S—— animately talking to a student who had come to take notes on his cases; without a moment's hesitation sister charged down the ward, they saw her coming; the student retreated to the nearest bed and seized a history card, Nurse S—— hurried round the opposite side of the block of fireplaces with sister in pursuit. I retreated to the bathroom, knowing that the finish of that episode would be enacted in that aristocratic quarter, and was calmly washing the basins round with tow and soft soap when the pursued and pursuer arrived. Sister's ruddy face was more highly coloured than usual, her protruding underlip was, if possible, more prominent than ever, I could see that she was extremely angry. "You are an exceedingly naughty probationer, and I shall certainly report you to matron; you know quite well you have no right to talk to the young men;" and so on until Nurse S—— was reduced to tears. The whole scene was quite a novelty to me. I must guard against the "young men."

Tuesday, 9.30 p.m.—Hurrah! to my little book. I went back to the male ward this morning; I felt I quite liked the silent, sad-faced Sister St. Margaret, when I got back to her after that dreadful old lady in the female ward. Even staff nurse seemed more genial, and condescended to teach me how to make a linseed poultice. A week to-day since I left home; it seems like a year, but I mustn't think about it or I believe I shall cry. There is home sister's voice: "Turn your light out, nurse."

### The Nurse's Never.

By a Medical Lecturer on Nursing.

(*Continued from page* 34.)

Never trust anything but abundance of fresh air as a disinfectant for a sick room. "Fumigations are absolutely essential: they make such an abominable smell that they compel you to open the windows."

Never be afraid of infection. "True nursing ignores infection except to prevent it. Cleanliness and fresh air from open windows, with unremitting attention to the patient, are the only defence a true nurse either asks or needs."

Never put a small square of carpet on a polished floor at the bedside. A ward is not a skating rink.

Never use carpets in a sick room if you can obtain matting.

Never use bed-curtains unless your patient particularly wishes them. All their advantages can be gained by using screens.

Never put a patient with pneumonia or bronchitis in a bed near a fire.

Never put a patient on a low or wide bed if you can get a high or narrow one.

Never use heavy bedclothing. Weight and warmth are not synonymous.

Never shake pillows or bedclothes over your patient. Always shake them away from the bed.

Never clean your furniture by laying out your clean clothes upon your dirty chairs or sofa, nor clean your walls or doors by hanging your clean gowns on them.

Never imagine that dust is removed from a room by simply distributing it more equally throughout the room.

Never make a drying-shed of your ward or sick room.

Never use your patient as a clothes-horse by piling up the bedclothes on him when making a bed. Place the clothes on a chair by the bedside.

Never let a patient read in bed with the light in his eyes. Put something white (paper or a screen) at the head of the bed to reflect the light.

Never allow an under sheet to become "rucky." If you do, you need not wonder that a patient feels uncomfortable, and gets bed sores.

Never turn all the bedclothes down in a heap when uncovering a patient for examination. Keep the upper sheet over him, and separate from the rest of the clothes.

Never stick pins into pillows or beds. You may stick them in a water-pillow or a water-bed some day, and then there will be trouble.

Never use a water-bed until you know whether it leaks or not.

Never put a patient on a water-bed until you have sat on it yourself, and have found that it buoys you up.

Never do your patient's hair up on the back of her head. Do it up in a plait on each side.

Never let a day pass without examining your patient for signs of bed-sores.

Never neglect the regular washing of your patient. Many diseases are more surely acquired by the skin than by any other channel.

Never use soap and cold water to clean your patient: use warm water.

Never use a clinical thermometer without knowing whether or not it registers correctly.

Never shake down the mercury immediately after using the thermometer, unless you have first placed the bulb of the thermometer in cold water.

(*To be continued.*)

### Compensation.

By a Nurse.

I lifted my thin white hands, and looked at them ruefully. Then with a painful effort I rose, and gazed earnestly at my own reflexion in the mirror over the mantel-shelf. Such a wan pinched faced looked back at me. For a moment I stared at it wonderingly, and then dropped wearily back into the cushioned chair.

Of course I knew I should be much changed. I knew I had been very ill, that my life had hung on a thread for weeks. Through those long, long nights of semi-conscious suffering I had been aware of my critical condition. Even before my fellow-workers, who had nursed me so tenderly, hid the temperature chart lest I should read, my own knowledge told me that the fever was very high. Everyone had been good to me. I should always remember their thoughtful kindness.

The matron's eyes had filled with tears of sympathy when she gave me the doctor's verdict—"I must not think of nursing again for 12 months, perhaps not then."

And it was all for the sake of a rough dock labourer, from whom I had taken the infection. Most likely he had started drinking again by now, and ill-treating the crushed little wife and half-starved children. It didn't seem worth the sacrifice, and I had meant to make the coming year so full of usefulness. There was another side to the question too. I should have to go home. And the warm loving welcome awaiting me could not alter the fact that my staying there would be a strain on my mother's limited income. I had so gloried in having combined a position of independence with a life work so full of noble interest. And now——

"These flowers have just been left for you with a note—there is no answer."

I thanked the wardmaid in a languid voice, and took the bunch of common autumn blossoms indifferently. Then I looked at the letter. It was soiled, and crumpled, and badly written.

"Dear Nurse,—I write these few lines to hope you are better, and to ask you to please accept the flowers. You don't know how badly we've all felt while you were ill. Tom, he's been to the Hospital gate every morning. He knows as you caught the fever off of him. Dear Nurse, I can never, never thank you for what you've done for us. Tom, he's a different man now. He's not been into a public since he's been back to work. And he says its through your goodness he's so changed. God bless you wherever you go. With the best respects and gratitude of Mary K."

### Presentations.

Holborn Infirmary.—The officers and nursing staff of the Holborn Infirmary have presented Dr. Thomas Evans, M.B.M.S., with a handsome silver spirit tantalus and Gladstone bag on his resignation in consequence of his promotion to the post of resident medical officer of the City Road Workhouse.

### To Nurses.

**We invite contributions from any of our readers, and shall be glad to pay for "Notes on News from the Nursing World," or for articles describing nursing experiences, or dealing with any nursing question from an original point of view. The minimum payment for contributions is 5s., but we welcome interesting contributions of a column, or a page, in length. It may be added that notices of entertainments, presentations, and deaths are not paid for, but, of course, we are always glad to receive them. All rejected manuscripts are returned in due course, and all payments for manuscripts used are made as early as possible after the beginning of each quarter.**

### Everybody's Opinion.

[Correspondence on all subjects is invited, but we cannot in any way be responsible for the opinions expressed by our correspondents. No communication can be entertained if the name and address of the correspondent is not given, as a guarantee of good faith but not necessarily for publication, or unless one side of the paper only is written on.]

### MISS CURETON'S TESTIMONIAL.

"The Hon. Secretary and Treasurer of Addenbrooke's Hospital, Cambridge," writes: May I give publicity through your columns to the fact that the fund for the testimonial to be presented to Miss Cureton, matron of Addenbrooke's Hospital, will be closed on the 26th instant? Friends desiring to subscribe to that fund are requested to forward their contributions either to me or pay them to the account at Messrs. Mortlock's Bank at their earliest convenience.

### LORD LINDLEY AND COTTAGE NURSES.

"M. W." writes: With reference to Lord Lindley's speech made at Hillingdon a short time ago, I see no reason why a cottage nurse should of necessity be a cottager. When illness falls upon the poor in villages they require nursing in just the same way as those in towns who are attended by the district nurse or in hospitals. A nurse who works in the right spirit, whether a so-called cottage nurse or not, would, I presume, go to the aid of suffering ones with the idea of doing all in her power for the care of her patient, not taking her own comfort into consideration, and consenting to do common things with high intentions. The cottage nurse, although not masquerading as a "trained nurse," has work to do which at times is by no means work for an amateur as many would be able to prove.

### WAR NURSES IN SOUTH AFRICA.

"A. N. S. R." writes from 21 General Hospital, Deelfontein: In reply to a note in The Hospital of September 7 saying that the War Office should send out an ample supply of nurses, I beg to state that there are more than sufficient out here at present, and that any nurse can (if she wishes it) go home at the end of a year's service out in South Africa. I have worked for the Army for nearly two years, and have had the greatest kindness and consideration shown me by the Army Superintendents and also by those in authority in the R. A. M. C. If the nurses are sick they are well nursed. At the commencement of the war the strain of course was very great; but then who was to know that there would be an outbreak of enteric amongst the troops? I must say that all the nurses look remarkably well out here, and I think if anyone is tired out it is the Army Superintendents, who have had the strain and worry of constantly changing nurses from the commencement of the war. The nurses who are out here and have been for some time know the climate, and if they have a pressure of work can stand it better than those fresh out from England.

### THE SCARCITY OF NURSES IN WORKHOUSE INFIRMARIES.

"Yorkshire" writes: May I add my experience (four years in one of our largest London hospitals and four years in workhouse infirmaries) to the many letters you have published on the subject of workhouse nursing; I quite agree with one of the guardians at Newcastle that until the working of the infirmary is quite separate to the workhouse there will always be friction. It is not likely that a nurse who has been fully trained and probably has testimonials from some of our most renowned doctors will brook the workhouse master and matron who have had no training. Surely if a woman is considered fit to be a superintendent nurse she is capable of seeing that the infirmary is kept as it should be, and that the nurses are trained and do their work. The motto of guardians generally appears to be "never trust a woman." Beside the workhouse master and matron the medical officer is deputed to see that the infirmary is kept clean, give the nurses extra leave, etc., which in a general hospital would be left to the matron, she, of course, always teaching them that the doctor is their superior officer and must be obeyed in everything. In the first infirmary I worked in the matron had entire control of the nursing staff and the life in every way was quite equal to the hospital. Since then and after my hospital training I have worked in two workhouse infirmaries where there has always been friction owing to the master and superintendent nurse not agreeing, and no one feels this more than the nurses.

"One who Loves her Work" writes: "A Poor Law Nurse" must have been landed in one of the workhouse infirmaries where they are very far behind the times. Her letter would frighten anyone from entering our ranks. May I give quite a different picture of at least one, and I know of several other infirmaries where there are nurses who are neither fast, flighty, drunken, lazy, ignorant nor underbred, but bright, cheerful, intelligent and capable. Such adjectives as "A Poor Law Nurse" uses are of the past, so far as my experience of eleven years goes. In the infirmary where I am now there are over one hundred patients, six nurses and one superintendent. There is not one of the nurses who takes alcohol in any form; it does not even enter the infirmary. As to being lazy, I have found during my experience only two nurses who were not more than willing to do everything they were asked; even staying in their off-duty time to help when busy. This is not a training school, but everything is made as interesting as possible by the doctor and the superintendent. The doctor will have things done up to the mark, and would not term such people as your correspondent speaks of nurses at all. Nor would he allow them to remain. We are taught midwifery, and have to do all the work for the patients who are sick or bedridden. We prefer to do all that is necessary ourselves, and not to get others to do it for us, which would not be allowed even if we wished it. One cannot help pitying "A Poor Law Nurse" who feels so sore, and I trust that she will soon be more fortunate in her experience.

"A Poor Law Nurse" writes: Another "Poor Law Nurse" has introduced an entirely different element in her letter in suggesting the separation of workhouses and infirmaries. I fully endorse all that she says, but I would point out that the necessity for this has been recognised for a long time; indeed, one of the best-managed infirmaries in London shook itself free from the workhouse as many as 20 years ago. "E. A.," in her indulgence in satire, has rather missed the point. I am delighted to read "Lady Superintendent's" excellent report of her infirmary. I sincerely congratulate her upon the splendid work she is doing, but I read between the lines that she agrees in the main with me, for she says: "The sisters or charge nurses have been trained in London and good provincial general hospitals, besides which I promote probationers of my own training." In this way she gets the "spirit" and "tone" of the general hospital into her own infirmary, and consequently she secures a good class of nurses and probationers, and I have no doubt has no difficulty in finding them. I would specially emphasise the fact that it is this "spirit," this "tone," which is so necessary in the infirmary. I also thank "Hospital Sister" for her letter. I agree with her that it should not depend on "the nurses' own efforts whether they get trained or not," but I repeat that in infirmaries it does so depend. When a woman enters a hospital as probationer, she does not know. She is ignorant of what her duties will be, of how much she is expected to learn; she is ignorant of her responsibilities, and does not know how to take advantage of her opportunities; nay, more, she does not know what these opportunities are. She may be without ideals or not, but in any case she has to have them strengthened, filled out, made more definite. Unless a woman has had plenty of experience, knows her own ignorance, and also how to get the most out of every small occurrence, she may go through an infirmary training and be almost untrained. I disagree with "Hospital Sister" when she says "that training in infirmaries can never be so good as in general hospitals." We get the advantage of having both acute and chronic cases, and the one kind of nursing needs just as much training as the other; indeed to keep clean and free from bedsores an old chronic hemiplegia or paralysis is a task which will tax the best of nurses trained in a general hospital. In some infirmaries the acute cases are kept in separate wards and the nurses take the chronic wards in turn. "Hospital Sister" is labouring under a misapprehension if she thinks that I imagine the L.O.S. makes a nurse. My remarks on that subject were merely to the effect that the opportunities for practical midwifery are not taken advantage of as they should be in infirmaries, and that if they were they would be such as a nurse in a general hospital would not have. I congratulate "P.S." and "An Infirmary Nurse" on their happy experiences. May they never have any other, and I hasten to assure "Infirmary Nurse" that it is with no inclinaton to "sneer" or "look down" on anyone that I have written as I have done. I received my own training in an infirmary, and it is my desire to raise the standard of these nurses that prompted me to write on the subject. Let us not blink facts nor try to hide the faults that we have. It is only by looking well into reasons for failure that we can ever achieve success; and if all infirmary authorities put their shoulder to the wheel we shall surely shove on the heavily-laden waggon that is now stuck fast in the slough of "a scarcity of Poor Law nurses."

### CROYDON INFIRMARY TROUBLES.

"Interested" writes: I doubt if the stocktaking is the only trouble at the Croydon Infirmary. The nurses are far from settled down yet. The winter session for lectures has now commenced and made more trouble. The senior probationers and juniors are all to be lectured, too, together, making one class. Of course, the nurses have always formerly been divided into three classes. But this year the lectures are to be different, just as everything else. This is causing no little strife among the nurses, as several will have finished their three years in a few weeks and are only having the same lectures as the juniors.

"Mrs. Latter"—formerly Miss de Pledge—a member of the Executive Committee and Registration Board of the Royal British Nurses' Association, writes: I, in common with many others, have followed the progress of the Croydon Infirmary dispute with mingled feelings of sorrow and disgust. Sorrow at the persecution and indignities to which a conscientious woman has been subjected, disgust at the methods employed to belittle her in the eyes of her subordinates, and through her the injury caused through the progress of Poor Law Infirmary nursing in this country. The action of the Croydon guardians in depriving the matron of her position as superintendent of nurses, can but recoil on their own heads, as no self-respecting woman would be likely to seek employment under a board which has shown itself so regardless of the interests of its nurses. Without a matron's signature the nurses' certificate is not worth the paper it is written on, and what is more, nurses know this, and are not likely to give up three years of their lives to obtain a document which is practically useless. The local Government Board is largely responsible for the unhappy state of things which too often exists in the matron's department. By their orders a matron in the present day is required to be a trained nurse and presumably a woman of refinement and education, and yet she is given no more authority, nor is her position any more defined in relation to the nursing staff than it was thirty years ago when trained nurses in the majority of infirmaries were unknown, and all that she was expected to do was to superintend the work of the scrubbers, and if need be assist in the kitchen. The matron's position is, therefore, so anomalous to-day, that it is not very surprising to find that the best women hesitate to take a post which is so absolutely dependent on the goodwill, or otherwise, of the medical superintendent. How completely this is the case is evidenced by the extract you publish in the last number of The Hospital of the medical officer's report to the guardians, and which is expressed in language hardly calculated to pour oil on troubled waters. That it should be possible for any officer to refer in such terms to a colleague, and that colleague a woman whose position as head of the nursing staff should be respected, is the gravest indictment which can be offered of the system that permits it. When will guardians learn that there may be another side to some of these questions—that the woman is not invariably in the wrong, though she may be too loyal or too proud to attempt to justify herself? If they could but realise that the tone of the female staff is regulated by the matron, and not by the medical superintendent—for women take their tone from a woman and not from a man, I venture to affirm, without fear of contradiction, for I speak advisedly, that if boards of guardians would apply for a new nursing order defining the duties and position of the matron, and so free her from those petty interferences that hamper her work, and impede the progress of nursing in Poor Law infirmaries, much of the present almost unavoidable friction would disappear. All honour to Miss Julian that she had the courage of her opinions, and declined to affix her signature to the certificates of nurses whom she considered unworthy. By this very act she proved her fitness for the post she holds, and that she was prepared to suffer for conscience' sake as she has done, shows her also to be a woman of whom the nursing profession may well be proud. It would have been so much easier and pleasanter for her to have signed the papers, and so have assisted to swell the ranks of unworthy members of the profession, but would she not have been guilty thereby of something very much like perjury?

### Appointments.

Ashton-under-Lyne Infectious Hospital.—Miss Mary Burwick has been appointed matron. She was trained at Leeds Infirmary, and has since been sister at Salop Infirmary; night superintendent at the Royal Infirmary, Aberdeen; matron of Bridgend Hospital, and matron of the Pembroke and Haverfordwest Infirmary.

Eye Infirmary, Bath.—Miss Annie B. Boyd has been appointed matron. She was trained at the Royal Eye Infirmary, Plymouth, and has also had experience in general work at the Princess Alice Hospital, Eastbourne, and Dr. Savage's Private Hospital, Birmingham.

Hahnemann Hospital, Liverpool. — Miss Gertrude Davis has been appointed lady superintendent. She was trained at Cardiff Infirmary. She has since been assistant matron in the same institution, and matron of Porth Cottage Hospital.

Paddington Green Children's Hospital.—Miss Ida Gissett has been appointed night sister. She was trained at Cheltenham Hospital, and Guy's Hospital, London. She holds the L.O.S. certificate.

Parish of Fulham Infirmary.—Miss Kate Nichols and Miss Elizabeth Moncrieff have been appointed sisters. Miss Nichols was trained at Birmingham Infirmary for three years, and has since been staff nurse at Birmingham Infirmary, and St. Mary Hospital, Paddington; superintendent nurse at Belper Union Hospital, and matron at the Nurses' Home, Newport, Mon. Miss Moncrieff was trained at St. Pancras Infirmary for three years, and has since been charge nurse at Bolton Union Hospital.

Southwark Infirmary, East Dulwich.—Miss M. L. Stone has been appointed sister. She was trained at the Royal Portsmouth Hospital, and has since been staff nurse for 18 months at University College Hospital. She has also done holiday duty at St. George's Hospital, London.

West Highland Cottage Hospital, Oban. — Miss Helen Reid has been appointed matron. She was trained at the Western Infirmary, Glasgow, and the Rotunda Hospital, Dublin. She has since been night sister at the Rotunda Hospital, Dublin, and staff nurse at the Longmore Hospital, Edinburgh.

### Echoes from the Outside World.

### AN OPEN LETTER TO A HOSPITAL NURSE.

The sensation of the week has been the removal by the Commander-in-Chief of Sir Redvers Buller from his command at Aldershot. This painful step is, undoubtedly, the result of what has been called "the blazing indiscretion" of General Buller in making a speech at Westminster which was contrary to the King's regulations. While everyone will regret that the career of a brave man has thus been brought to a premature close, few will be disposed to question the judgment of the Commander-in-Chief, who is far more prone to err on the side of leniency than on that of severity. Both in South Africa and at home, the further announcement that Sir John French has been appointed to the command of the First Army Corps will be received with great enthusiasm, especially since the gallant general is to remain in South Africa so long as his valuable services are needed.

We have been so accustomed to hear and to read of air-ships, which, according to their inventors, would do marvellous things, and which when tested always came ignominiously to grief, that most of us had begun to think the time for aerial success was not yet. Therefore the triumph scored by M. Santos Dumont came upon us as a considerable surprise. This is by no means the first time that the young Brazilian has made the attempt to secure the Deutsch prize, but some untoward event has always prevented his obtaining the coveted 100,000 francs (£4,000) which had been offered to the man who, starting from the park of the Aéro Club, could conduct his aerial conveyance round and over the Eiffel Tower and return to his starting place—a distance of seven miles—in less than half an hour. The committee had become so convinced of the difficulties of the task, now that the still summer weather had passed, that when M. Santos Dumont telephoned he was going to make an attempt again on Saturday afternoon, there were only five members present. Though he started badly the first time and had to do it again, and though once or twice it seemed as if the aeronaut must be dashed to pieces, he accomplished his journey without accident in the given time. But it is a little doubtful, owing to a technicality—the guide rope had not been seized the moment the balloon crossed the club ground, and the committee maintain that it should have been—whether after all he will receive the money, which he intended to divide between those who helped him and the poor of Paris. Though the sail through the air was safely accomplished, and all due honour should be accorded to the patient, persevering, and clever inventor of the cigar-shaped machine which flies by the aid of a motor, the time is yet far distant when we shall take an air-ship to cross London as naturally as a tram car. The dangers are still enormous, and the rolling is said to be so great that sky-sickness would probably be as troublesome as *mal-de-mer*.

It is difficult to understand that in a Christian land the fact of a white man having asked a black man to his house to dinner, could be denounced as "a blunder worse than a crime," and as "the most damnable outrage ever perpetrated by any citizen of the United States." Yet these are the opinions expressed by two of the Southern newspapers of America in commenting upon the news that the Rev. Booker Washington, the well-known negro educationalist, had dined with President Roosevelt at the White House. The eminence of the professor apparently counts for nothing against the prejudice of the Southerners, and he is undoubtedly an eminent man, for, born a slave without a name, when he obtained his freedom as a lad he had everything to learn and none to give him a helping hand. Yet to-day as a scholar many white men are his inferiors. But according to some, because he has a black skin, he should be placed for ever on a platform immeasurably below the man fortunate enough to have been born with a white one. The "crime" has certainly not been committed by the President through thoughtlessness. His action is the outcome of his determination to show that he has the courage of his convictions, and that a Christian and a gentleman should behave as such. It has since been asserted that Mr. Roosevelt has seriously prejudiced his chance of being re-elected at the end of his present term of office, but those who know much of his character will not be surprised to learn that he refuses to permit considerations of this kind to weigh with him in the selection of his guests at the White House.

The experiment which is to be made in Battersea at the end of this year, should be especially interesting to all women, more particularly, perhaps, to maternity nurses. The Borough Council of Battersea has been much impressed by the appalling mortality among infants, which stands at present at the high figure of 162 per 1,000 for children under a year old. This is believed to arise principally from improper "bottle" feeding. To remedy this evil it is proposed by the Battersea Municipality to supply humanised and sterilised milk to the poor of the district at 1s. 6d. a week. For this sum six hermetically-sealed bottles containing four ounces apiece are to be obtained each day. All that will have to be done by the mother is to put the bottle in hot water to warm and then give the contents to the child. There is only enough for one meal in one bottle, therefore cleanliness is ensured, more especially as there is no tube, the turning of the stopper revealing the means by which the child can draw the milk. The mothers will be asked to bring their babies once a fortnight in order that they may be weighed, and that it may be seen whether they are making such progress as they ought to make. It is hoped also by this means that some influence may be brought to bear on mothers whose offspring appear to be neglected in the matter of cleanliness. The experiment, I am glad to say, is to be conducted on business, not on philanthropic, lines. £600 will be needed to fit up the necessary premises to provide a steriliser and furnish the plant required to humanise the milk, which is coming direct from a big dairy farm in the country. But after this outlay there should be little further expense to the ratepayer. It is true that if only 100 infants a week are fed there will be a loss of £50 a year on the transaction, but it is hardly probable that so few parents will take advantage of the proposal to supply for less than 3d. a day the best food possible for their children. If 200 a week are fed the undertaking will clear its expenses and 400 will secure a profit. Surely it is not rash to predict that when the institution is really known amongst the poor the profit will be forthcoming.

Until the last few years, men have been apt to hurl the reproach at women that there was one thing of which they were incapable—they might write a good novel but they could never write a good play. Latterly, however, two or three triumphs have been scored by women, notably by "John Oliver Hobbes," whose smart sayings seem to have caught the public taste. But now a yet more wonderful thing has happened. The Playgoers' Club, out of the efforts of 400 competitors, has selected the play written by a lady as the best, and it is to be staged in triumph with two actor-managers in the cast. The strangest thing is that Miss Netta Syrett admits that she is not a playgoer and really knows nothing of the theatre-going world. She has written novels for some years, and has always been considered rather good at dialogue. She began "A Modern Love Story" as a novel, but finding that the plot lent itself much better to the stage than to a book, she altered it with the happiest results. The scene is laid in London and in the Riviera, and though naturally Miss Syrett refuses to divulge any other details, she admits that she has been tremendously interested in the works of Ibsen. Therefore, it is safe to predict that the story will be up to date. The lady who has just scored such a success is a many-sided genius. She was educated at the North London Collegiate School for Girls in the days of Miss Buss, became a high-sohool teacher in Swansea, but gave it up after two years, and now divides her time between writing novels, such as "Nobody's Fault" and "The Tree of Life," publishing fairy stories like "The Garden of Delight," and lecturing on English literature at schools in and around London. Her young pupils are said to be intensely excited at the striking success which their teacher has achieved.

### A Book and its Story.

### A REAL HEROINE.*

Maxwell Gray's latest novel is a gracefully told love story, slight in construction, but written with the refinement and force that characterise her compositions. The story "goes" from start to finish, in spite of a reasoned discursiveness at times that diverts the reader's attention from the central theme, and quickens by suspending interest in the fate of an unusually interesting hero and heroine, Hugh Beaumont, soldier, *sans peur et sans reproche*, and Marcia Ludlow, a woman of character and temperament.

Marcia's personality is a strong one, and in her whole-hearted devotion to her lover she is a real heroine. With good looks and a magnetic attractiveness that draw men and women alike, all unconsciously, into the circle of her influence, she yet remains alone in the isolation of soul that is the fate of those so gifted, until they meet the one and only love to whom their heart goes out intuitively, and then too often comes sorrow and disaster.

This is the thread from which the romance of "Four Leaved Clover" is woven. Not an everyday romance by any means, for Marcia and Hugh Beaumont are not an everyday man and woman, and it is this that holds the reader through the uncertainties of a particularly unsmooth running of true love's course. Eight years before the story opens Marcia had met Major Beaumont at her first dance, and he had given her a four leaved clover for luck. "From that day her life was one romantic poem, and no man was more than comrade to her because of him." Beaumont goes abroad and distinguishes himself in the Soudan, and they meet unexpectedly in the dusk of an autumn afternoon, when Marcia, tired from a hard day's riding, comes into the shadows of the hall of her uncle's country house, accompanied by her cousin Jack. Here she stands out before the reader. "The glowing cheeks, shining eyes, and laughing lips, that could be full of pain, were eloquent now of health and thought, and the swing of the supple figure was not without power. . . . She was tall, with the grace of a slender shape, and long limbs well brought out by her riding habit. The hall was cosy and homelike, with a table drawn up before a bright fire and screens and well-padded lounges invitingly grouped about. A hanging lamp threw a soft, but hardly sufficient light on the region about the fireplace." It had been suggested that Marcia's cousin, pretty Mab Tyndall, had arranged the old hall in this cosy and inviting manner to decoy willing victims into prolonged *tête-à-tête* interviews and "the light of a guileless face with perfectly-cut features and innocent appealing eyes emerging from the shadow of a settle, a screen, a palm or a press, and followed by a more or less sheepish-looking male countenance was a familiar one to her brother Jack." Marcia called her "La belle dame sans Merci." On this particular afternoon Major Beaumont suddenly appears "from the ends of the earth," as Jack Tyndall explained and, not aware that he and Marcia had met before, introduced him as "our old friend," the effect upon her is so great that she stands as if in a dream. "The laughing lips were white, the deep eyes lustrous; she looked as if she were trying to speak but could not. . . . Beaumont seeing Marcia sway came from behind Mabel to help her; but before he could reach her she had collapsed and sunk on the floor at his feet." Since their casual meeting the two had not met for eight years. Marcia in the meantime had developed into a beautiful woman. She had been to India and back again to join her brother, and had seen much of life and society. Beaumont had carried with him no vision of her face in his wanderings, as she had of his. Her sudden faintness was put down to over-fatigue in the hunting field. Jack Tyndall, who had been out with her all day, naturally enough settles the cause, and adds, "Girls are so foolhardy; they will try and do as men do, and they can't."

"I never care to see ladies in the hunting-field," Beaumont rejoined; "they are better at home."

The following day, as Marcia sits at her window, she watches Hugh Beaumont ride away, and she, still under the spell of his presence, sees before her "a face with dark eyes, a spare straight figure, and hears a voice, a laugh, certain words that repeat themselves," such small things as can fill a mind and furnish food for fancy and reflection for a long, long time. "She sat in the embrasure of the upper window to witness this departure, sat long in the sunshine with wet eyes and a dreamy face, always looking at the interlaced shadows the trees threw on the grass-edged gravel, seeing and hearing more than the mere robin's song, and the caw of the busy rooks." "Marcia, among other devoted followers, has a soldier lover whose attentions are both persistent and repugnant to her. She perfectly understands the man, and is quite unmoved by his apparent devotion. He, on his part, finding his case hopeless, determines to ruin her chance of happiness with Beaumont. He owes her a grudge for having put him to open shame upon one occasion when she finds him, under the plea of enforcing obedience, beating one of his setters with such savage cruelty that the poor brute is half dead, and on the spur of the moment, "when she saw Borman with white, ugly face and glittering eyes, and the poor creature bleeding at his feet, a mist came over her eyes, fire flashed through her brain, and before the uplifted rod had descended again—she never knew how—Borman was caught unexpectedly between the eyes and . . . sent backwards heels over head into the thick mud pond." This ignominous position for an officer in Her Majesty's army was converted from a tragic, to a comic one, by the onslaught of a flock of cackling geese who approached the helpless flounderer with fluttering wings and hissing beaks. To revenge this insult to outraged vanity he pursues a course which only a man of utterly despicable nature could have taken. The scene in which Marcia and Hugh Beaumont meet as the result of a forged letter purporting to come from Beaumont to Marcia, is a really fine piece of writing and quite the best scene in the book. She receives the declaration of Beaumont's love contained in it with joy, but some misgivings. Nothing in his manner has ever led her to divine by the light of love given but not returned, that his feeling for her was more than friendly interest; but she answers it and promises to meet him, as it suggests, in the park early the following morning. And there she finds him awaiting her. The pathos and dignity of the trying situation when Beaumont finds that Marcia has been the dupe of an unprincipled scoundrel, is beyond praise. "The place of meeting was on mossy ground starred with wild flowers and dropped chesnut bloom. . . . She saw the hyacinths swaying in the grass and heard the cuckoo's morning cry before she looked up, as women look with such feelings as hers in their hearts, and perceived that his face was pale, his eyes grave, his mouth a little stern. . . . "As I live I have written you no letter, dear lady, I cannot deceive you." From this sad point the story increases in power and interest until it ends happily to the sound of their marriage bells.

* "Four-leaved Clover." By Maxwell Gray. (Publisher: W. Heinemann, London. 1 vol. 6s.)

### For Reading to the Sick.

"WHEN DAY'S SHADOWS LENGTHEN."

When day's shadows lengthen,
Jesu, be Thou near;
Pardon, comfort, strengthen,
Chase away my fear;
Love and hope be deepened,
Faith more strong and clear.

When the night grows darkest
And the stars are pale,
When the foe assembles
In death's misty vale,
Be Thou Sword and Helmet,
Be Thou Shield and Mail.

. . . . .

Blessed Warfare over,
Endless Rest alone.

*Rev. F. Lee.*

Who that has really suffered has not felt that in gazing upward towards the Prince of sufferers, all things become changed in their relations? The melancholy past merges into the present, and the present becomes lost in a future—a future of hope, a future of mercy, a future that swallows up all sorrows, stills the cry of all anguish, deadens the edge of all pain. There with Him is all that we have lost, and all that we have mourned for; there the loved ones that have gone before; there the innocent joys of childhood that soon fleeted by; there the quick sympathies that soon were checked; there the warm affections that soon grew cold; there the fair hopes to which disappointment brought blight and decay. All are with Him. And to Him, if our hearts yet remain true to God and to our better selves, every suffering only tends to bring us nearer and nearer. We gaze only the more earnestly there, where we know we shall find all: "Where our treasure is, there shall our heart be also."

*Bishop Ellicott.*

The heavens will never grow dark above us in this life, while we guard in our hearts the grace of pity: and after this life we may go out into the mystery that lies beyond with His own clear word of promise: when we need it perhaps most terribly, we shall find most richly the pity which has been with us all along: for it is the beatitude of the pitiful that they shall obtain pity.—*Francis Paget.*

Lord of my nights and days!
Let my desire be,
Not to be rid of earth,
But nearer Thee.

If I may nearer draw
Through lengthened grief and pain.
Then to continue here,
Must be my gain.

Sorrow's long lesson o'er,
Death's discipline gone through,
Thou wilt unfold to me
What Joy can do.

Lord of my heart and hopes!
Let my desire be,
Not to be rid of earth
But one with Thee.—*C. M. Noel.*

### Notes and Queries.

**The Editor is always willing to answer in this column, without any fee, all reasonable questions, as soon as possible.**
**But the following rules must be carefully observed:—**
**1. Every communication must be accompanied by the name and address of the writer.**
**2. The question must always bear upon nursing, directly or indirectly.**
**If an answer is required by letter a fee of half-a-crown must be enclosed with the note containing the inquiry.**

*Answer.*

I think if B. L. will apply to the editor of the *Quiver*, she will find that that magazine gives, or did give, medals for long service. About two years ago I was nursing in a family where three maids and one manservant had them for different lengths of service, ranging from 25 years to 65 years. I am not sure, but I believe that they also had certificates as well.—*N. L.*

*Idiot Boy.*

(30) Will you kindly tell me of some home where a little idiot boy, of four, could be placed? His parents can afford to pay a small sum.—*Nurse Maude.*

He may be eligible for the Darenth Schools. Apply to the clerk, the Metropolitan Asylums Board, Victoria Embankment, E.C., or at the Earlswood Asylum, Red Hill.

*The New Army Nursing Service.*

(31) I see in the account of "Queen Alexandra's Imperial Military Nursing Service" that one of the conditions of admission to the service is that a "candidate . . . must possess a certificate of not less than three years' training and service in medical and surgical nursing in a civil hospital recognised by the Advisory Board." Will you kindly tell me if the training of the Bristol General Hospital would be recognised by the Advisory Board?

The recommendations of the Committee have not yet been confirmed by Royal Warrant.

*Home Nursing.*

(32) I am a district nurse (Queen's), and have been asked to read a paper on Home Nursing to a large class of young women. I should like to comply with the request, knowing that it would be useful, but not having undertaken anything of the kind before, I should be glad if you could advise me of any small book or simple lectures bearing on home nursing.—*A. W.*

You could not do better than obtain the handbooks issued by the St. John's Ambulance Association on "First Aid" and "Nursing." For large-sized illustrations, "The First Aid Designs," just published by Messrs. John Wright and Co., Stonebridge, Bristol, might be useful.

*Congress of Tuberculosis.*

(33) Can you kindly tell me where I can get a full report of the Congress on Tuberculosis, and the price?—*An Invalid Nurse.*

The official report is not yet issued.

*Treatment by Light.*

(34) I should be grateful if you could tell me if there is an inexpensive treatise on the treatment of lupus by "light." Any information on the subject will be welcome.—*A. B.*

"Phototherapy," by Professor Niels Finsen, Copenhagen, translated by Dr. Sequeira (Edward Arnold, 27 Bedford Street, Strand).

*Lupus and Rodent Ulcer.*

(35) 1. Will you kindly tell me if lupus and rodent ulcer belong to the same family of disease? 2. Is rodent ulcer a milder form of lupus? 3. Is rodent ulcer infectious or contagious, and what is the best way to disinfect a patient's bed linen?—*A. S.*

1. No. 2. No. 3. Rodent ulcer is often septic, and the bed linen is to be treated in the same way as that of any other septic case. It is not contagious in the sense of its infection producing rodent ulcer.

*Address.*

(36) Will you kindly give me the address of the Army Nursing Reserve in London?—*G. M.*

18 Victoria Street, S.W.

### Standard Books of Reference.

"The Nursing Profession: How and Where to Train." 2s. net; post free 2s. 4d.
"Burdett's Official Nursing Directory." 3s. net; post free, 3s. 4d.
"Burdett's Hospitals and Charities." 5s.
"The Nurses' Dictionary of Medical Terms." 2s.
"Burdett's Series of Nursing Text-Books." 1s. each.
"A Handbook for Nurses." (Illustrated). 5s.
"Nursing: Its Theory and Practice." New Edition. 3s. 6d.
"Helps in Sickness and to Health." Fifteenth Thousand. 5s.
"The Physiological Feeding of Infants." 1s.
"The Physiological Nursery Chart." 1s.; post free, 1s. 3d.
"Hospital Expenditure: The Commissariat." 2s. 6d.
All these are published by the Scientific Press, Ltd., and may be obtained through any bookseller or direct from the publishers 28 and 29 Southampton Street, London, W.C.

### Travel Notes.

By Our Travelling Correspondent.

### LXXXIV.—IN GRASSE FOR SIX WEEKS.

It is only a short time ago—safely, indeed, within the treacherous memory of the middle-aged—that Grasse was a little-known primitive town far removed from the all-searching eye of the tourist, and then to my mind it was perfection. Perhaps it betrays a surly disposition, but I must confess that I like to be fairly free from my compatriots when I am on the Continent; one does not travel far for the pleasure of still enjoying the society of Miss Brown and Mr. Jones. To encounter different types of the human species is quite as refreshing as to see mountains and lakes instead of Fleet Street and Clapham Junction. Even now Grasse is not so very popular, partly, probably, because it is some distance from the sea and accommodation (unless you are content with humble native inns) is dear and scarce.

### The Journey to Grasse and its Climatic Advantages.

The journey is rather expensive, as is always the case to the South of France—£10 2s. 4d. first return and £7 6s. 1d. second, ditto. This is by Newhaven and Dieppe. By Calais and Dover it is £11 19s. and £8 12s. 9d. You would not, however, be likely to take a return ticket, for those going to Grasse at all would probably stay several months. The cheapest single tickets are respectively £6 10s. 10d. and £4 10s. 8d.

There are very considerable advantages to be gained from a stay in Grasse. Its situation is nigh 1,230 feet above sea-level, which prevents it from being relaxing, which otherwise it might be from its extremely sheltered position. There are many who cannot stay at Nice, Mentone or Cannes, because, from the state of their nervous system, the near proximity of the sea is unsuitable, who would find Grasse quite a haven of refuge. Supposing that the visitor is not really in ill-health, but simply desiring to benefit by the floods of sunshine to be absorbed in Grasse, he would do well at the Hôtel Muraour, where terms are very moderate, but it is too much in the centre of the town to be quite suitable for those who are undeniably invalids.

### The Perfumery Industry.

This is really colossal and most interesting to outsiders. The most of our really good scents come from Grasse, and the proprietors of the different factories are most kind and courteous in allowing visitors to inspect their preliminary operations. Acres and miles of ground around the town are covered with flowers, which are not only turned into the distilleries and produce the most exquisite perfumes, but are also grown for the purposes of making a delicious sweetmeat. It was, I believe, Monsieur Negre who first conceived the idea of utilising flowers in this way, and his Parma violet sweetmeats, which are the pure blossoms thrown into boiling syrup, are now of world-wide renown.

The two processes of scent-making, known as the "hot" and "cold," were a great astonishment to me, and gave me quite new ideas on the subject. The delicious Parma violet perfume, so popular with us all, is made by soaking the petals in hot pig's fat.

It is a delightful experience on a dewy morning to walk through several acres of the violets, which are planted where they shall receive some shade from trees, for they are too delicate to stand the full force of the southern sun.

### Sights of Grasse and the Neighbourhood.

There are great opportunities in the old town for the sketcher and photographer; the quaint streets and dark nooks afford endless variety of subjects, but beyond these charms Grasse has no antiquarian resources. There are, however, numerous excursions to be made in every direction. It is only 12 miles to Cannes. Since the opening of the Sud railway Nice is reached in two ways, either by the main line *via* Cannes or by the new railway *via* Colomars, one of the prettiest bits of line in Europe. If you cycle, a very charming excursion may be made by train to Colomars, about 28 miles, and thence by road to Nice, another 11 or 12. It is an ideal run, and I am very much surprised that the facilities offered by the Sud railway are not more widely appreciated.

Fourteen miles east from Grasse lies Vence, reached either by road or rail. A delightful old place; it seems out of all reason that so sleepy and silent a town should have a station. Once a bishopric it has a cathedral; its distinguishing feature is a sumptuous choir gallery which runs round three sides of the church instead of being confined to the West end. The miserere seats are fifteenth-century work and form a delightful study. We picnicked outside Vence on a breezy but warm April day in such a situation; the lovely Alpes Maritimes all spread around us of a vivid yet iridescent blue and little rock towns visible here and there—that immediately facing us I took to be St. Paul.

From Grasse to Cagnes by the Corniche is from 11 to 12 miles and perfectly lovely. I walked one way returning by rail. This exquisite road is now almost deserted in favour of the rail which is such a saving of time and expense. Cagnes is another old-world place in spite of its station; the Grimaldi family have one of their fortified castles in the midst of the town and see it from what point you will it is always picturesque. From Cagnes to St. Paul (well worth seeing) a diligence runs which is a comfort to non-cyclists.

Villeneuve-Loubet is another charming rock village on the road between Grasse and Cagnes. Its position is very striking, the houses climbing up the steep sides of a hill whose summit is crowned by a frowning and massive castle with a singular five-sided tower. Here, too, we picknicked, this time like tramps by the roadside, as I could not tear myself from a sketch I was making of the castle and the road into the valley. The making of our tea from the celebrated tea-basket proving such an enthralling spectacle to the natives that we were soon surrounded by an interested and sniffing crowd. I do not remember ever having suffered under such persistent and aggressive sniffing anywhere in my many wanderings.

There is a short walking excursion, which I am sure you would enjoy, to the mountain village of St. Jeannet; you can either take the train direct to St. Jeannet or, what is more interesting, get out at Vence and walk four miles on the side of the mountain. If you cannot walk thus both ways you might return direct by rail. There is a charm about these hill villages that exercises a great fascination over me, and I find that most people when once they know about them are equally charmed. Next week I shall take you in spirit still further up in these little-known lateral valleys north of the Riviera. The road runs generally almost by the side of the Sud railway, and naturally if you are a cyclist or a good walker the road is the better way; but I must say the trains show no indecent haste, and I have even been able to make a sketch whilst our guard or engine-driver has been enjoying the charms of conversation with the dwellers in the wilds.

### TRAVEL NOTES AND QUERIES.

Boarding House at Harrogate (Miss H.).—I fear there is nothing cheap at Harrogate. There are the hydropathic establishments which are very popular, and more reasonable than the hotels. These are "The Cairn," "The Harlow Manor," "The Spa," etc. Of boarding houses at moderate rates I have no knowledge. In your place, I should go for a night to the "People's Temperance Hotel," in Albert Street, then look about for yourself. Nothing is cheap in the season, which is now over till next May.

